# Electrohydrodynamic Techniques for the Manufacture and/or Immobilization of Vesicles

**DOI:** 10.3390/polym15040795

**Published:** 2023-02-04

**Authors:** María Celina Sánchez-Cerviño, Codrin Paul Fuioaga, Leonard Ionut Atanase, Gustavo A. Abraham, Guadalupe Rivero

**Affiliations:** 1 Research Institute of Materials Science and Technology, INTEMA (UNMdP-CONICET), Av. Colón 10850, Mar del Plata B7606BWV, Argentina; 2 Faculty of Medical Dentistry, “Apollonia” University of Iasi, 700511 Iasi, Romania; 3 “Cristofor Simionescu” Faculty of Chemical Engineering and Environmental Protection, “Gheorghe Asachi” Technical University of Iasi, 700050 Iasi, Romania; 4 Academy of Romanian Scientists, 050045 Bucharest, Romania

**Keywords:** vesicles, electrospinning, electrospraying, drug delivery systems

## Abstract

The development of accurate drug delivery systems is one of the main challenges in the biomedical field. A huge variety of structures, such as vesicles, nanoparticles, and nanofibers, have been proposed as carriers for bioactive agents, aiming for precision in administration and dosage, safety, and bioavailability. This review covers the use of electrohydrodynamic techniques both for the immobilization and for the synthesis of vesicles in a non-conventional way. The state of the art discusses the most recent advances in this field as well as the advantages and limitations of electrospun and electrosprayed amphiphilic structures as precursor templates for the in situ vesicle self-assembly. Finally, the perspectives and challenges of combined strategies for the development of advanced structures for the delivery of bioactive agents are analyzed.

## 1. Introduction

The development of accurate drug delivery systems is still a main challenge in biomaterials science. Over the last decades, countless structures have been proposed as carriers for bioactive agents, aiming for precision in administration, dosage, safety, and bioavailability. Vesicles, nanoparticles, and nanofibers are some of the many systems largely explored to this end. This review covers the application of electrohydrodynamic techniques for both the manufacture and the immobilization of vesicles. Although the electrospinning and electrospraying techniques are often chosen for their ability to load agents (or smaller supramolecular structures) within nano/microfibers or particles, their potential to fabricate vesicles in a non-conventional way is still underexplored. Herein, the advantages and limitations of electrospun and electrosprayed amphiphilic structures as precursor templates for the in situ vesicle self-assembly are discussed. The review of some recent advanced examples supports the fact that the combination of different structures and manufacturing techniques allows obtaining materials with improved functionality for drug delivery applications. 

## 2. Vesicles

### 2.1. Supramolecular Systems

**Figure 1:**
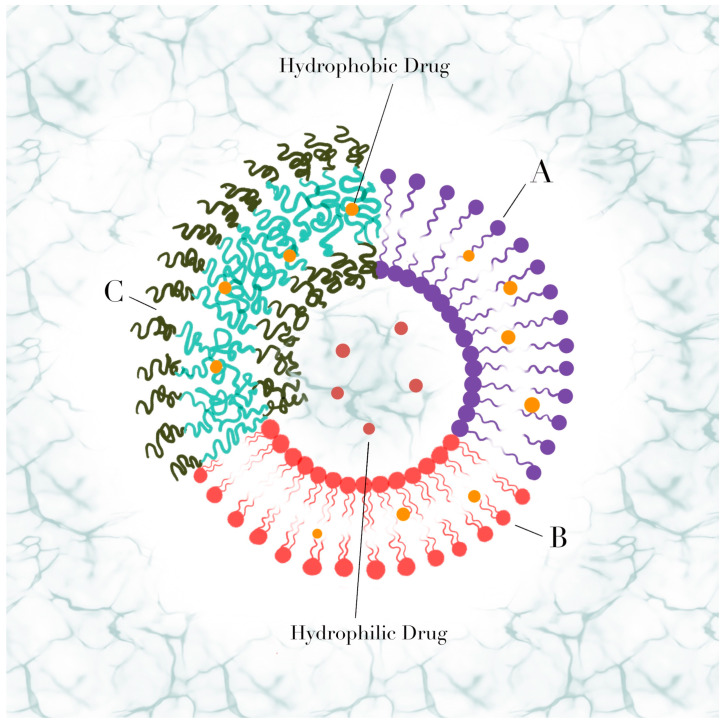
Comparative scheme of different vesicle structures: (**A**) niosomes; (**B**) liposomes; (**C**) polymersomes.

Vesicles are self-assembled spherical molecular aggregates composed of one or multiple bilayers of amphiphilic molecules that surround an aqueous compartment. The hydrophobic region is composed of saturated or unsaturated hydrocarbon chains and has functional groups that are soluble in water. Many studies have shown that the molecular shape of the amphiphilic molecules determines the supramolecular structure type that is formed when they self-assemble (e.g., vesicles, micelles, etc.). Indeed, the size of the hydrophobic and hydrophilic regions in the amphiphiles must be similar for obtaining vesicles. In an aqueous medium, vesicles are formed as a result of non-covalent interactions between the hydrophobic regions and the interactions of the hydrophilic regions with the medium. Vesicles’ potential as nanocarriers in controlled drug-release systems is due to the ability to load hydrophilic substances in the internal aqueous phase and hydrophobic drugs or agents in the bilayer [1]. Depending on the type of amphiphilic molecules forming the bilayer sheets, vesicles (Figure 1) can be classified as liposomes (when artificially prepared from lipids), polymersomes (from synthetic block copolymers), or niosomes (from nonionic bilayers). Regarding the number of bilayers, vesicles are described as multilamellar (MLV) or unilamellar (ULV). In turn, ULV composed of one bilayer can be sorted based on their size, as giant (GUV, 1–100 µm), large (LUV, 100–1000 nm), or small unilamellar vesicles (SUV, 30–100 nm) [2].

### 2.2. Vesicles Fabrication Methods

Vesicles are metastable systems whose type and size depend on the fabrication processing type [3]. All existing methods can be classified into two types: solvent-free or solvent-displacement methods. When using solventless methods, amphiphiles are hydrated in an aqueous medium, and there are no organic solvents present in the vesicular solutions. In contrast, organic solvents are often used to first dissolve the amphiphiles, which are then placed in an aqueous medium prior to the organic solvent removal [4].

The main methods for obtaining liposomes are mechanical dispersion, reverse phase evaporation, solvent dispersion, and detergent removal [5]. The most widely used mechanical dispersion method is the thin film hydration method (TFHM). Shortly, the mixture of lipids is dissolved in a suitable organic solvent or mix of solvents (e.g., chloroform and methanol) in a flask where the hydrophobic drug may be added. In the second step, the solvents are evaporated in an inert atmosphere, and a thin film is formed. Next, the film is hydrated with an aqueous solution that may contain the hydrophilic drug of interest to be encapsulated. As the formed liposomes are MLV with high polydispersity, their size can be scaled using other additional methods, such as extrusion or sonication, in order to increase the homogeneity and reduce the size. However, the drugs or lipids can be degraded during this process, and the sonication probe may cause metal contamination. Although both hydrophobic and hydrophilic drugs can be encapsulated at the same time, this method uses toxic solvents and requires multiple steps. The reverse phase evaporation method is a variation where the dissolved lipid is mixed with an aqueous buffer (that may contain the hydrophilic drug), leading to the formation of an emulsion. Then, the organic solvent is evaporated by reducing pressure or heating. The mean vesicle size and polydispersity can be further reduced using additional methods. Solvent dispersion methods use a suitable organic solvent (e.g., ethanol, methanol, ether) to dissolve the lipid mixture and the hydrophobic drug, and the obtained solution is then injected into an aqueous phase with a syringe. The aqueous phase is preheated above the phase transition temperature of the lipids, and the organic solvent is evaporated under a vacuum or by heating above the boiling temperature. The aqueous phase could be pure water or a buffer containing a hydrophilic drug. By controlling the process parameters, small liposomes with low polydispersity can be obtained without additional homogenization steps. Drawbacks of these methods are the contamination with organic solvents, low loading rates, and exposure to high temperatures that may reduce the stability of lipids and active agents. The detergent removal method uses a mix of surfactants of high critical micelle concentration and lipids that are dissolved in a suitable organic solvent. The solvent is evaporated, and a thin film is obtained, which must be further hydrated with an aqueous solution (that contains the drug molecules). Next, the surfactant is removed by dialysis, gel filtration chromatography, hydrophobic bead adsorption, or dilution in order to formulate unilamellar vesicles. The disadvantages of this method include the loss of the hydrophilic drug when the detergent is removed and the detergent residue in the liposomes [2,6].

Conventional liposome processing methods are tedious and inefficient because of the many precise steps that affect the size, stability, and functionality [5,7]. Freeze-thaw cycles are often used as a conditioning technique to improve the encapsulation efficiency and laminarity of liposomes. Additionally, freeze-drying techniques (lyophilization) have been used to improve the stability of the final product for delivery applications [2,6], as well as new methods based on heating and microfluidics [7]. In all cases, the liposome manufacturing methods affect their properties, such as size, polydispersity, lamellarity, etc. Average size is a key quality assay as it affects loading efficiency, mucoadhesion, toxicity, biodistribution, safety, and cellular uptake (essential for in vivo applications) [8]. Many liposome-obtaining methods have also been applied to niosomes and polymersomes [4,9,10]. Table 1 lists a selection of processing methods to obtain different types of vesicles.

### 2.3. Advantages and Limitations of Active Agents’ Delivery Systems

Liposomes, which are safe and biodegradable, have many structural advantages based on their resemblance to biological membranes, which facilitates their interaction with cells, endocytosis, and fusion. Moreover, liposomes have great versatility to encapsulate active principles with different solubility. Hydrophobic drugs incorporated in the lipid bilayer often increase their bioavailability, while hydrophilic drugs can be loaded in the aqueous core. As controlled release systems, liposomes reduce side effects, minimizing the necessary dose and, therefore, the accumulation of drugs at the administration site. They provide passive targeting via enhanced permeability and retention effect in tumor area [12]. Furthermore, the surface of liposomes can be functionalized with specific ligands in order to direct the liposomes to a site of action, thus assuring the targeted therapy [13,14,15]. Nowadays, there are marketed liposomal formulations for cancer treatment, fungal treatment, photodynamic therapy, and pain management. Moreover, there is a large number of clinical trials for new applications around the world [6]. Many strategies have been developed to achieve a longer circulation of liposomes in the blood, such as adding polyethylene glycol (PEG) to the membrane. In addition to controlling the release of drugs, it increases its durability by providing protection from external agents, such as light, humidity, and oxygen [16]. The incorporation of PEG molecules bearing specific functional groups (amine, thiol, etc.) in the liposome surfaces is often intended to optimize their functional properties for specific applications [17]. However, PEGylated liposomes have been reported to reduce the interaction with target cells [18] or trigger unexpected immunoreactions [19]. On the downside, dispersions containing liposomes are unstable, and vesicles can aggregate, depending on the formulation. Their sensitivity to degradation and the complex formulation and functionalization steps are the main disadvantages limiting liposome applications [20].

Polymersomes, such as liposomes, can transport both hydrophobic and hydrophilic drugs [21]. However, their chemical versatility allows tuning the diffusion properties and easily modifying their surface, achieving specific and controlled release. In addition, a thicker membrane enables the storage of a greater amount (approximately 10 times more) of hydrophobic therapeutic agents. Despite this, they are not yet available on the market as the official approval is pending, and their industrial production in a monodisperse form of nanometric size is difficult [20]. Niosomes resemble liposomes, but due to their composition, they are not susceptible to oxidation or hydrolysis. They are stable, biocompatible, cheaper, and easy to store and handle. Niosome-based formulations are often easily reproducible as well. While the first uses were in the cosmetics industry, the sterilization process required for drug delivery application is a limiting factor. Heating sterilization methods are destructive when overcoming the transition temperature leading to drug leakage. Additives are often added to niosomes formulations to stabilize the dispersions, thus avoiding aggregation, fusion, and drug loss. Although the components of niosomes are generally considered innocuous, there are still some safety concerns, given that specific studies of the niosome toxicity after administration in animals have not yet been performed [9,16,22].

## 3. Electrohydrodynamic Techniques 

### 3.1. Fundamentals and Versatility

Electrospinning technology is a well-known electrohydrodynamic process that has attracted huge attention as a powerful and versatile processing technique for producing electrospun nanofibrous non-woven scaffolds/membranes [23,24,25]. Originally studied as a technology for drawing nanofibers from polymer solutions, the electrospinning process can be extended to obtain polymer-based nanocomposites, ceramic nanofibers, solid-to-porous nanofibers, solid-to-hollow nanofibers, as well as random to uniaxially aligned nanofibers [26,27]. Remarkably, electrospinning technology has progressed from fundamental concepts to relevant industrial applications, being one of the most popular sub-micro/nanofabrication processes [26,28,29,30,31]. Electrospun fibrous materials find many interesting emerging applications in several bio/nanotechnological research areas (biomedical, pharmaceutical, clothing, and healthcare [32,33,34,35,36]), including the fabrication of scaffolds for tissue engineering [37,38,39,40,41,42], bioactive agent delivery platforms (cells, gene, growth factors, or drugs)[43,44,45], wound healing [46], biosensors [32,47,48], and others [49,50].

Nowadays, electrospinning is applied for manufacturing one-dimensional nanostructures with extremely high surface area-to-volume ratio and high tunable porosity, topography, and morphology [51]. Moreover, electrospun nanofibers have unique properties due to flexibility in processing and the possibility to control the nanofiber composition in order to achieve the desired properties and surface functionalization. This versatility also extends to the chemical composition and to a wide variety of materials, such as synthetic/natural polymers, polymer blends, and polymer-ceramic or polymer-metallic nanocomposites, solutions, suspensions, emulsions, or melts. In addition, the high potential of electrospinning improvements for mass production of nanofiber-based products is currently the most relevant advantage [30,52].

The working principle of the technique is based on the electrostatic attraction of charges produced by the application of a very high electrostatic force by means of a high-voltage power source to a viscoelastic fluid (solution or melt). At a critical voltage value, a charged microjet of fluid overcomes the surface tension forming a cone-like droplet (Taylor cone) which is ejected from a nozzle. The generated microjet is accelerated towards a grounded collector, experimenting with whipping or bending motions as a result of uneven distribution of charges. The solvent is rapidly evaporated during the jet trajectory, and, at the same time, stretching and thinning of the fiber occur. Depending on the polymer molecular weight as well as the experimental and processing conditions, the microjet can be atomized into solid micro/nanoparticles or beads (a process known as electrospraying), or it can form a continuous jet that results in electrospun fiber deposited on the collector [51,53]. Electrospinning setups are highly variable, but the simplest and basic instrument consist of five components: a fluid reservoir (typically a syringe); a single blunt nozzle or spinneret; a syringe pump for controlling a constant flow rate of the solution or melt; a high-voltage power supply; and a grounded collector (or oppositely charged) placed at certain distance [51]. The process is governed by a set of operational parameters, which can be classified as intrinsic (solution properties), processing, and environmental parameters [54,55]. Solution properties include composition, viscosity (which depends on the polymer concentration and solvent), electrical conductivity, surface tension, and boiling point of the solvent. Processing parameters, such as operating voltage, needle tip-to-target distance, and flow rate, have a strong influence on the process. Among the environmental parameters, temperature and relative humidity deeply affect electrospinning, depending on the chemical structure of the polymer. Both alter the evaporation rate, thus changing the fiber diameter and morphology [56,57,58].

### 3.2. Nanofibers and Nanoparticles as Delivery Systems of Bioactive Agents

Nanofibrous scaffolds are highly valuable and attractive vehicles for targeted and localized drug delivery systems, which include active biomolecules (peptides, genes, and many other therapeutic agents) [36,59]. Flexibility in the selection of materials, active agents, and drug-loading methods allows the control of delivery mechanisms and release kinetics, hence tailoring the release process for each application.

Polymer-related parameters, including polymer composition, crystallinity, and molecular weight, also greatly affect drug release from nanofibers. Hydrophilic and amphiphilic copolymers result in increased drug loading capacity and decreased chances of burst release [43]. Polymers with high crystallinity degrees present slower release rates as compared to amorphous ones due to very low water uptake in highly ordered crystalline regions. Fiber diameter and alignment are also parameters that affect drug release. Smaller fiber diameters exhibit faster release due to a decreased distance for drug diffusion and a larger surface area. In addition, randomized fiber patterns are associated with faster drug release because of increased water uptake.

On the other hand, drug-related parameters that affect release from nanofibers include drug loading methods and content, the physical state of the drug, solubility, molecular weight, and drug–polymer interactions. A wide variety of electrospinning and post-processing treatments allow tailoring strategically-designed electrospun mats [60,61]. Active agent loading determines the release profile, and higher loads usually lead to faster release. Drug location is also a key factor for achieving the desired behavior. Several strategies for drug incorporation were explored and implemented: (i) Solution blending involves active agents or precursors dispersed throughout or mostly at the fiber surface. This is a conventional strategy for the production of monolithic fibers, i.e., fibers with a solid homogeneous matrix across the fiber diameter; (ii) Coaxial electrospinning allows better control of release profiles by suppressing the initial burst release. The encapsulation of sensitive biocompounds in a core surrounded by a polymeric shell protects them from being denatured in harsh or organic solvents or unfavorable environments [62]. The use of concentric needles that separate solutions in different channels leads to core/shell or three-layered nanofibers or nanoparticles [63]; (iii) Emulsion electrospinning enables the loading of active agents achieving, under certain conditions, a core/shell morphology [64]. In these cases, core/shell composition, architecture, layer thickness, and integrity play an important role in the sustained drug release process [65]. Moreover, post-processing electrospun mats by chemical or physical attachment (adsorption), dip-coating, or layer-by-layer assembly constitute other strategies to provide functionality and drug carrier capability for tuning the release kinetics [66,67]. Other factors that influence drug release behavior are the crystallinity and molecular weight of drugs. Crystalline drugs tend to be deposited onto the nanofiber surface, inducing burst release, while amorphous drugs remain inside and are released in a sustained way. As expected, low molecular weight drugs are mostly released faster than drugs with high molecular weight.

### 3.3. Limitations and Current Challenges 

Although the electrospinning technique and setups are relatively simple, similar results for the same material cannot be obtained unless the complete set of experimental conditions is constant. Thus, the basics of the electrospinning process, the relationship between solution parameters and processing, and the obtained fiber morphology have been widely investigated. Despite being a highly attractive and promising tool for obtaining fibrous scaffolds with desirable properties, there are several limitations that still require further analysis. Typically, as-spun nanofibers form two-dimensional structures of tightly compacted fibers with certain morphological and nanotopographical features. However, cellular organization and interactions require pore sizes that favor cell infiltration into the inner regions, thicknesses larger than a few hundred micrometers, and truly 3D complex structures for specific needs. Abel et al. [68] recently reviewed certain combinations of electrospinning with other techniques for the fabrication of 3D polymeric/composite nanofibrous scaffolds with improved cellular interactions. In addition, the use of green techniques constitutes another big challenge in the design of advanced manufacturing scaffolds. Solvent-free electrospinning, melt electrospinning writing, and techniques involving non-toxic solvents are being explored to produce precise and controllable hierarchical 3D structures with or without drug loading.

Electrospraying has proven to be an attractive and versatile tool for obtaining mono-dispersed particles by tuning applied voltage, flow rate, viscosity, and polymer concentration in the semi-diluted entangled regime [53]. However, these interrelated variables affect size, shape, and morphology, and they should be properly set to ensure batch-to-batch reproducibility. Moreover, results have sometimes been reported without detailed experimental conditions making it difficult to reproduce them. There are still numerous challenges to overcome for the preparation of core-shell drug-loaded particles and complex structures in a single-step process in a controlled way when using two or multi-needle systems. The lack of suitable properties in certain materials, such as biopolymers or ceramics, requires the use of additives that might not be biocompatible. Although these electrohydrodynamic techniques possess limitations and challenges, increasing research makes it an active and flourishing field.

## 4. Immobilization of Supramolecular Structures within/onto Electrospun/Electrosprayed Materials

### 4.1. Nano-in-Nano Systems 

The combination of different structures, in turn generated by diverse manufacturing techniques, is often desirable to achieve complex morphologies with functional advantages. Electrosprayed and electrospun structures can host bioactive agents alone or be included within smaller structures. Loaded nanostructures have been incorporated inside submicrometric carriers produced by the electrohydrodynamic techniques for several reasons:

- Protection: when the bioagent can be degraded/modified by the carrier chemical nature or the carrier matrix is not capable of providing an adequate barrier with the environment. Given that their structural functional groups are susceptible to chemical changes, natural bioactive compounds, such as polyphenols, vitamins, alkaloids, flavonoids, terpenoids, fatty acids, proteins, peptides, probiotics, etc. from different sources, require certain protection in order to maintain or improve the physicochemical functions and bioavailability [69]. Sometimes, a pre-confinement stage in smaller structures allows multicomponent extracts and phytocomplexes to preserve a synergistic effect with functional benefits.
- Location: if there is a requirement for the bioactive agent to be located in precise sites of the carrier structure (surface, core); then, a smaller nano-object can contribute to the manufacture of a specific architectural design. Nano-structures can be adsorbed, absorbed, or embedded in nanofibrous meshes due to the intrinsically interconnected porosity of electrospun membranes. However, more efficient attachments require stronger intermolecular or covalent bonding; thus, more complex synthetic strategies can be involved in the design.
- Delivery trigger: The design foresees a specific sequence for supplying the bioactive agent, as consecutive diffusion barriers, “smart” matrices sensitive to externals stimulus, etc. The use of polymers sensitive to temperature, pH, magnetic/electric fields, humidity, etc., for the manufacture of electrospun/electrosprayed host materials, allows triggering the delivery in an “autonomous” way. Some matrix materials can undergo phase transitions or selective dissolution patterns induced by temperature (e.g., PNIPAM) or specific environmental acidity changes (e.g., Eudragit^®^ series).

All criteria were exploited by Sonzogni et al. in the development of an enteric nano-in-nano protein delivery system [70]. Firstly, the agent was loaded into nanogels in order to increase treatment effectiveness while reducing adverse effects. Despite offering excellent beneficial water retention, the gel porosity does not protect the cargo. The loaded nanogels were then immobilized into the core of electrospun coaxial nanofibers. The use of a polymer with pH-selective dissolution as the sheath of these fibers ensured the delivery in the duodenum [70]. Saiding and Cui [71] have systematically reviewed some recent hybrid platforms composed of electrospun nanofibers containing different nanoparticles for biomedical applications. Other supramolecular systems, such as micelles, vesicles or liposomes, dendrimers, nanogels, etc., are often used to increase the drug accumulation [72]. As for all hybrid and composite materials, the main challenges lay in an appropriate and homogeneous filler distribution in the fiber/capsule polymeric matrix. Preventing agglomerations with precise incorporation allows exploiting the benefits derived from the large aspect ratio in the combination of nano-structures.

### 4.2. Nanofibrous Matrices and Submicrometric Capsules Containing Vesicles

The combination of vesicular carriers and polymeric scaffolds could generate composite materials with improved functionalities. While vesicles are more versatile carriers, their immobilization inside/over a scaffold locates them nearer the target area (than when a parenteral administration is used). Thus, hybrid systems may overcome the limitations of conventional vesicles/liposomes and upgrade their performance in terms of stability, delivery precision, and bio-accessibility.

Different systems based on liposome-loaded scaffolds have been recently reviewed for tissue engineering applications [12], particularly for bone regeneration [73]. Electrospun materials often provide the mechanical support required for scaffolds. Vesicles are mainly incorporated into electrospun fibers in two different ways: by surface modification or as internal load inside the core. Table 2 displays a selection of works from the last decade, where the versatility of strategies for the incorporation of vesicles/liposomes to electrospun nanofibers and nanoparticles is highlighted for different research fields.

**Figure 2:**
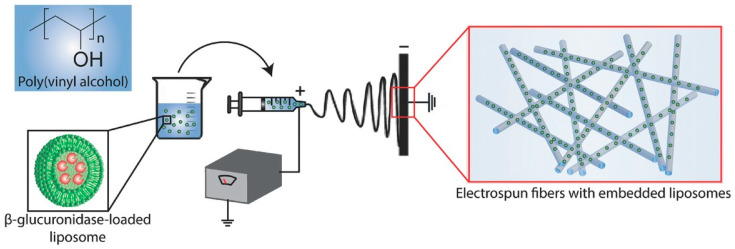
Scheme of assembly of electrospun polymeric fibers with embedded liposomes containing enzymes. Reproduced from [77] with permission from Creative Commons Attribution License.

If the loaded supramolecular structures are included within the fibers, the most frequent approach involves the mix of the (loaded) suspensions with the polymer solution prior to electrospinning. Agents include dyes, model drugs, enzymes, extracts, essential oils, and proteins, among other bioactive agents, with a foreseen functional activity in very diverse fields, ranging from food packaging to biomaterials for tissue regeneration or cancer therapy. In some cases, the main goal is to improve system stability. For example, Chandrawati et al. incorporated enzymes into electrospun fibers inside liposomal sub-compartments in order to protect proteins against deactivation [77] (Figure 2). Frequently, the design of the systems points to tuning the release kinetics (to extend it or to slow it down by weakening a burst effect). For both purposes, coaxial electrospinning, co-electrospinning, and microsol-electrospinning variations were also implemented. Mickova et al. [87] compared the performance of a single (blend) and coaxial electrospinning for encapsulating liposomes. Core–shell liposome-enriched nanofibers were accomplished to preserve the aqueous environment inside intact liposomes, leading to better stabilization and viability of biologically active compounds for tissue engineering. In general, improvements in the safety, handling, storage, and active use of materials have been achieved by embedding liposomal vesicles in electrospun nanofibers.

A synergetic effect is sometimes simply achieved by placing together both the fibers and liposomes in the cell co-culture [91]. Otherwise, covalent immobilization or grafting techniques are mainly chosen to effectively attach the supramolecular structures to the fiber surface. In turn, the lipid composition can be susceptible to modification with characteristic functional groups or targeting ligands to promote specific cell interactions. The spatial organization achieved by surface modification usually intends to generate a specific action at the local level (vectorized release, environmental responsiveness, biological functionality, etc.). In some cases, the drug is loaded inside the liposomes instead of the fibers in order to avoid the potential damage caused by high voltage electrostatic field during electrohydrodynamic processing [12].

Similarly, the electrospraying technique has been employed for the production of encapsulated liposomes or vesicles within particles. Gomez Mascaraque et al. reported a significant increase in stability and bioaccessibility of curcumin when entrapped in liposomes (prepared with the injection method) and then coated with whey protein by electrospraying for food-grade applications [79]. They also managed to improve solubility and slow down the curcumin release from electrospun fibers [83]. Instead, acetyl curcumin-loaded core/shell liposome nanoparticles prepared by electrospray process were designed for theranostics, and delivery applications and the release profiles were studied in detail [90].

## 5. Electrohydrodynamic Techniques as Non-Conventional Vesicle Fabrication Method

Conventional electrohydrodynamic processing allows producing sub-micrometric fibers and particles. However, a novel combined strategy uses electrospun/electrosprayed materials as templates for the fabrication of vesicles. In this way, electrohydrodynamic techniques are a tool for obtaining smaller supramolecular structures overcoming the dimensional limitations of the techniques. Liquid vesicle dispersions may reach a more intimate contact with cellular membranes in target tissues, given the permeability provided by their amphiphilic nature.

Hybrid polymeric solutions containing phospholipids are first electrospun/electrosprayed to fabricate solid templates. When required, these precursors allow obtaining vesicles by in situ self-assembly when dissolved in water. As a manufacturing procedure, this innovative method enables the manipulation of components’ molecular arrays and offers important advantages for vesicle storage, transport, and administration. These benefits positively affect their functionality, stability, and bioaccessibility as bioagent carriers. Besides chemical affinity, vesicle self-assembly requires both the transport and direct contact between building blocks. With conventional methods, this step is carried out by small-scale agitation of viscous solutions, multiple stages of sonication or extrusion, or other complex methodologies that can leave residual solvents.

Furthermore, vesicles are often obtained as aqueous dispersions where long-term stability is a critical factor [96]. These drawbacks can be overcome by pre-confining the components by nanofabrication using electrohydrodynamic techniques. The rapid solvent evaporation during processing leads to solid structures where components maintain the physical state of the pristine fluid. When dissolved, the vesicle’s self-assembly takes place since components are already pre-localized in microenvironments. In this way, “top-down” electrohydrodynamic techniques produce sub-micrometric materials that originate nano-objects by “bottom-up” self-assembly when in contact with water. Nanofibrous or particulate precursors can be stored until usage in a solid state as templates in order to generate liquid suspensions of vesicles by dissolution, which do not require sterilization.

### 5.1. Confinement within Solid Submicrometric Precursors: Key Parameters and Versatility

**Figure 3:**
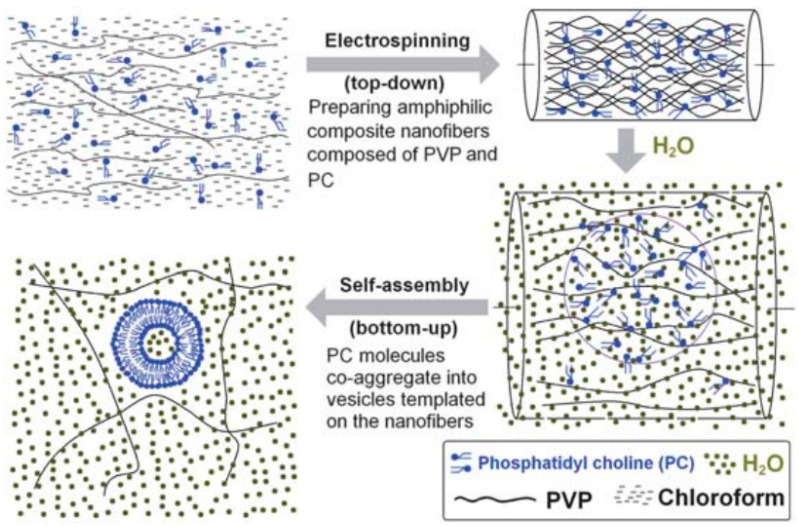
Schematic diagram illustrating the self-assembly of liposomes from PVP-PC electrospun nanofibers. Reprinted with permission from [97] the Royal Society of Chemistry; permission conveyed through the Copyright Clearance Center, Inc.

**Figure 4:**
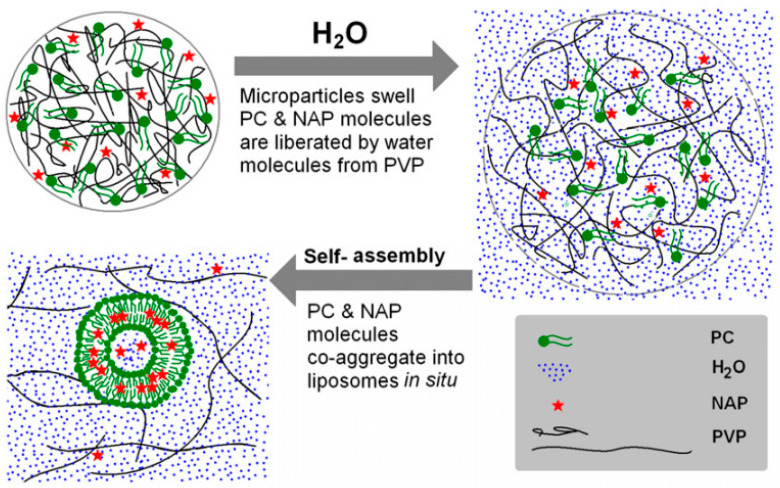
Schematic diagram illustrating the self-assembly of liposomes from microparticles based on PVP-PC, containing naproxen (NAP) as a loaded drug agent. Used with permission of IOP Publishing, Ltd., from [96]; permission conveyed through the Copyright Clearance Center, Inc.

Yu et al. first produced self-assembly liposomes from amphiphilic electrospun nanofibers and electrospun microparticles. The use of poly(vinylpyrrolidone) (PVP) as a hydrophilic matrix in nanofibers [97] or microparticles [96] with variable ratios of phosphatidylcholine (PC) allowed to manipulate the resulting liposomes sizes as a function of phospholipids concentration. Chloroform was used as a solvent in both cases for the mixtures of phosphatidylcholine and K60 and K17 PVP, respectively. In the case of amphiphilic fibers, as PC content increased (between 9 and 33%), the fiber diameters decreased (in the range of 740–580 nm). On the contrary, when dissolved in water, the diameters of the self-assembled liposomes linearly increased (120–269 nm), and the polydispersity index diminished (Figure 3). On the other hand, naproxen was incorporated in the microcapsule-based templates (960 nm diameter average), leading to liposomes with a 91.3% encapsulation rate that later freed 80% of the drug by diffusion in 24 h (Figure 4).

Regarding the vesicle size control, Matsuura et al. [98] reported the production of hollow phosphorylcholine polymer vesicles of 300–400 μm obtained from coaxially electrosprayed CaP/chitosan microparticles templates with diameters of 280–500 μm. Interestingly, the vesicle diameter increased with larger inner diameters of the inner needle of the coaxial electrospray system. Living yeast cells were successfully encapsulated within these hollow polymer vesicles.

Other works explored different polymeric matrices for the precursors, with varying phospholipids ratios, in order to provide the systems with sensitivity to temperature, pH, or magnetic fields. Jin et al. [99] used temperature-sensitive poly(*N*-isopropylacrylamide) and PC in chloroform/ethanol as pristine fluids for the preparation of electrosprayed microparticles. The phase transition temperature of the resulting liposomes was adjusted by varying the phospholipid content. While two populations of large unilamellar vesicles can be detected at 25 °C (142 and 955 nm), a single one is distinguished at 37 °C (278 nm), with a marked reduced polydispersity index. As a model drug, ketoprofen was incorporated in the formulation, and the release studies evidenced a precise temperature dependence. The same model drug was incorporated (with 75% encapsulation efficiency) during the fabrication of electrosprayed microparticles based on Eudragit L100 and PC, with chloroform/*N,N*-dimethylacetamide solvent system [100]. When dissolved in buffer solution, distinct release profiles as a function of temperature were obtained from the resulting loaded liposomes.

Song et al. [101] incorporated Fe_3_O_4_ nanoparticles into the mixture of polymer and PC prior to electrospinning. Magnetic liposomes were obtained when the electrospun fibers were dissolved in water for 12 h after centrifugation and freeze-drying. In this case, liposome sizes could be controlled by varying the oxide content.

### 5.2. Recent Drug Delivery Systems

In terms of functional performance, recent works have reported interesting results of drug-loaded liposomal systems based on electrosprayed templates. Chen et al. [102,103] used a self-assembly liposome-based system based on electrospun hybrid fibers for the administration of carvedilol in the buccal mucosa. Multilayered fibrous prototypes were proposed for the delivery of drugs with a high first-pass effect by using chitosan/PVA or cellulose acetate/ carboxy methyl cellulose as a mucoadhesive layer. Results showed improvements in permeation and bioavailability, drug load, shelf life, and liposome integrity, as well as beneficial features in terms of production technology and store conditions when compared with the analogous liquid suspensions. The PC ratio and polymer molecular weight significantly affected the drug encapsulation efficiency.

Collier et al. [104] proposed a variation of the methodology based on a one-step fabrication of doxorubicin-loaded-liposomes via coaxial electrospray. Buffers and lipids were separately pumped, and self-assembly was claimed to occur when the two phases met. A liquid buffer collector contained the remote loadable drug. When comparing the release profiles, electrosprayed liposomes retained the cargo longer than their thin-film hydration counterparts. Although liposome sizes and encapsulation efficiency had similar results (70–72%), the platform envisaged interesting benefits for scaling up production. Duong et al. [105] proved that electrospray remote loading achieved better final loading for liposomal resiquimod, an antiviral and antitumoral drug.

Haemanthamine, an anticancer agent, was loaded into amphiphilic nanofibers (197–534 nm diameter) intended for a solid template for self-assembled liposomes (63–401 nm diameter). The platform aimed to enhance the chemical stability and efficacy of the alkaloid in potential anticancer drug therapies when administrated via a parenteral route [106].

Laidmäe et al. [5] compared the structural features, efficiency, and drug-release behavior of liposomes loaded with chloramphenicol antibacterial drug produced after hydration of electrospun amphiphilic nanofibrous templates and with traditional manufacture methods as counterparts. Multilamellar liposomes, with smaller sizes but lower encapsulation efficiency, were obtained from fibrous–solid templates. Finally, Sánchez Cerviño et al. [107] have recently explored the effect of different solvent systems, pre-homogenization steps, and coaxial processing of the hybrid lipid–polymeric pristine fluids on the resulting vesicle size and structure. Furthermore, the differences in the lipid nature resulted in vesicles with varied permeability to probe molecules.

## 6. Perspectives and Challenges

Electrohydrodynamic techniques are widely spread technologies for the immobilization of bioactive agents or smaller loaded supramolecular structures. The incorporation can be made during processing by including them in the pristine fluid or as a post-processing surface modification. Vesicles are spherical systems formed by self-assembled amphiphilic molecules with concrete benefits to encapsulate and deliver both hydrophilic and hydrophobic agents. Vesicular systems as drug carriers have achieved better bioavailability and interaction with cell membranes leading to some marketed formulations with clinical use in the healthcare sector. However, the immobilization of vesicles onto/inside electrospun fibers or electrosprayed particles enabled the development of materials with superior properties in terms of stability, agent protection, and controlled release.

With a different approach, electrohydrodynamic techniques have been proposed as a non-conventional vesicle manufacturing method. The dissolution of electrospun/electrosprayed amphiphilic precursor templates in water triggers the in situ vesicle self-assembly, leading to small supramolecular structures with low polydispersity. This strategy is promoted to be extremely easier and cheaper than the current vesicle manufacture technologies allowing the storage of the solid precursors so that the vesicular dispersions could be prepared when required. However, long-term stability studies are still pending. Despite the interesting recently reported systems with loaded drugs for specific applications, further work is required in order to elucidate the involved mechanisms and correlate the compositional and electrohydrodynamic processing parameters with the structural characteristics of the resulting vesicle dispersions.

## Figures and Tables

**Table 1:** Selection of main vesicle preparation methods.

Methods	Liposomes [6]	Niosomes [9,11]	Polymerosomes [4,10]
Thin-film (thin-layer) hydration	x	x	x
Reverse-phase evaporation	x	x	
Solvent injection	x	x	x
Detergent removal	x		
Dehydratation-rehydration (sonication)	x	x	
Supercritical fluidic	x		
Microfluidics	x	x	x
Heating-based	x	x	
Freeze and thaw	x	x	
pH jumping	x		
Bubble-based		x	
Polymerization-induced self-assembly			x
Solvent-switch			x
Emulsion phase transfer			x
Transmembrane pH gradient drug uptake		x	
Electroformation			x

**Table 2:** Recent works reporting different strategies for the incorporation of loaded vesicles/liposomes to electrospun nanofibers or electrosprayed nanoparticles for diverse goals and application areas. Sorted by date reported.

Design	Loaded Active Agent	Polymer Matrix	Application	Goal	Reference
Loaded vesicles or liposomes mixed with solution prior electrospinning	5-Fluorouracil and paeonolum (anticancer model drugs)	PEO	Drug delivery	Dual hydrophilic/hydrophobic drug-delivery system	[74]
	β-carotene	PVA, PEO	Food	Protection of antioxidant activity (photostability); solubilization	[75]
	Cinnamon essential oil/β-cyclodextrin loaded proteins	PEO	Antibacterial food packaging	Stimulated release by proteolysis	[76]
	β-glucuronidase enzyme	PVA	Enzyme prodrug therapy	Stabilization for sustained biocatalysis	[77]
	Eugenol (+ SO_2_ nanoparticles)	PEO	Antioxidant food packaging	Improved stability	[78]
	Curcumin	Whey protein	Food applications	Stability, bioaccessability	[79]
	Calcein	PVA	Tissue engineering/drug delivery	Increased stability	[80]
	Tea tree oil	Chitosan	Food packaging	Improved antibacterial property	[81]
		Chitosan/PEO	Antimicrobial material	Sustained release	[82]
	Curcumin/green tea extract	Gelatin/zein	Food packaging	Improved solubility, slowed-down release	[83]
	Rhodamine-B (dye)	PCL	Tissue engineering	Controlled delivery	[84]
Loaded liposomes mixed with the core polymer solution prior coaxial electrospinning	Rhodamine B	PVP and hyaluronate	Wound healing	Stability	[85]
	Naproxen (NAP)	Cellulose acetate; hyaluronate	Wound dressing	Extended drug release	[86]
	Horseradish peroxidase (model protein); growth factors	PVA, PCL	Tissue engineering	Stability of biologically active compounds	[87]
Loaded liposomes mixed with polymer solution prior co-electrospinning (simultaneous)	Epigallocatechin-3-gallate	Gelatin/PCL	Skin regeneration	Antioxidant activity	[88]
Loaded nanoliposomes encapsulated into the core layer of core-shell nanofibers by microsol-electrospinning	Lysyl oxidase-like 1 plasmids	Polylactide-co-PCL/hyaluronicacid	Tissue regeneration	Local accumulation and biological availability	[89]
Phospholipid precursors and polymer nanoparticles mixed prior electrospraying to give core/shell particles	Acetyl curcumin	PLGA	Drug delivery	Sustained release	[90]
Mixture of loaded conjugated PEG-liposomes and loaded electrospun fibers, in cells co-cultures	Resveratrol (in fibers) + siRNA (in liposomes)	PCL/gelatin	Cancer treatment	Dual delivery devices approach with two drugs (different cellular pathways)	[91]
Application of liposomal formulations over electrospun nanofibers	Nano-copper, silver and gold	PCL	Targeted delivery systems (cosmetics, medicines)	Anti-bacterial and antifungal property	[92]
Covalent immobilization of loaded liposomes at the surface of electrospun fibers	BMP-2 peptide	Poly L-lactic acid	Bone tissue engineering	Sustained release	[93]
	Dexamethasone	PCL	Bone tissue engineering	Local release	[17]
Plasmid-loaded cationic liposomes grafted in the surface of microsol core-shell electrospun fibers	Interleukin-4; nerve growth factor	Amino polylactic acid /hyaluronic acid	Nerve function recovery	pH-responsive delivery + sustained release	[94]
Grafting of loaded polyethylene glycol acrylate liposomes to the electrospun fibers<	Deferoxamine	Gelatin-methacrylic anhydride	Tissue regeneration (bone)	Local delivery	[95]

## Data Availability

Not applicable.
